# Telehealth and people with disabilities in the United Kingdom: a scoping review

**DOI:** 10.3389/fpubh.2025.1504318

**Published:** 2025-02-12

**Authors:** Mezhen Ko, Matthew Azzopardi, Constantinos Loizou, Abison Logeswaran, Benjamin Ng, Agata Pacho, Yu Jeat Chong

**Affiliations:** ^1^Modality LLP, Birmingham, United Kingdom; ^2^Department of Health Services Research and Policy, London School of Hygiene and Tropical Medicine, London, United Kingdom; ^3^Moorfields Eye Hospital, London, United Kingdom; ^4^Christ Church, University of Oxford, Oxford, United Kingdom

**Keywords:** telehealth, telemedicine, healthcare accessibility, disability, remote healthcare, healthcare equality

## Abstract

**Introduction:**

Telehealth, also sometimes known as telemedicine, is the use of communication technologies to deliver healthcare remotely, has become increasingly vital, particularly since the COVID-19 pandemic. While telehealth can improve healthcare access, it may exacerbate inequities for people with disabilities. This scoping review explores the needs, experiences, and difficulties people with disabilities face when accessing telehealth services in the United Kingdom’s (UK) National Health Service (NHS).

**Methods:**

A systematic search was conducted using the PRISMA for Scoping Reviews (PRISMA-ScR) guidelines. The search terms included variations of “telehealth,” “disability,” “impairment,” “United Kingdom,” and “NHS.” Studies published after January 2010 were included if they addressed the experiences of people with disabilities when using telehealth. Ten studies met the inclusion criteria, and findings were synthesized into five key themes: patient and carer satisfaction, benefits of telehealth, healthcare provider perspectives, disability-specific barriers, and technological barriers.

**Results:**

The studies highlighted varied experiences across different disabilities, telehealth technologies, and medical specialties. While patients and carers generally expressed satisfaction with telehealth’s convenience and accessibility, a preference for face-to-face consultations remained. Key barriers included technological challenges such as poor internet connectivity, unfamiliarity with digital tools, and device access, as well as disability-specific challenges, particularly for sensory impairments. Reported benefits of telehealth included improved access to care and flexibility for patients with disabilities. However, healthcare provider perspectives highlighted concerns about the ability to build a rapport and perform thorough assessments remotely.

**Conclusion:**

Telehealth should complement traditional care through a hybrid approach. Future efforts must focus on improving technological accessibility, training healthcare providers, and co-designing solutions with patients to promote equitable healthcare access for people with disabilities.

## 1 Introduction

Telehealth, also known as telemedicine, is the delivery of healthcare services remotely through the use of communication technologies (1). The concept has evolved significantly since its first documented use in 1879, when an anonymous writer described a doctor diagnosing a sick child over the telephone – an invention that had just been introduced by Alexander Graham Bell (2). Over time, telehealth has evolved from simple telephone consultations to more advanced digital platforms, spurred by the rise of internet and mobile technologies. Recognizing telehealth’s potential to overcome geographical barriers in healthcare, especially in underserved regions, in 2005 the World Health Organisation (WHO) encouraged member states to enhance their information and communication technology (ICT) infrastructure, in order to ensure equitable, affordable, and universal access to healthcare services (3, 4).

The past decade has seen remarkable advancements in technology and significant reductions in the cost of communication devices and internet services, greatly expanding telehealth’s scope and applications (5). The COVID-19 pandemic in 2020 further accelerated the adoption of telehealth solutions, particularly within the National Health Service (NHS) in the United Kingdom (UK) (6, 7). By March 2020, telemedicine accounted for approximately 10% of outpatient appointments in the NHS, a sharp rise from 3.5% just a year earlier (8). To support this shift, the NHS rolled out the ‘Attend Anywhere’ platform for video consultations nationally, resulting in nearly 80,000 remote consultations conducted by May 2020 (9).

While the surge in telehealth usage has improved healthcare access for many, it also carries the risk of deepening the existing inequities experienced by people with disabilities (10). Disabilities encompass a wide range of physical, sensory, mental, or intellectual impairments that can profoundly impact individuals’ daily lives (11). Globally, over one billion people live with disability, including approximately 11 million in the UK (12, 13).

Evidence suggest that individuals with disabilities often experience poorer access to healthcare, including primary and cancer care, due to various challenges such as difficulty attending appointments and the lack of reasonable adjustments (14–16). Telehealth has the potential to address these barriers by allowing individuals with various impairments to receive care from the comfort of their homes. This approach empowers individuals by giving them control over their health management, while reducing the logistical challenges of physical travel (17). However, if telehealth solutions are not designed with the needs of end-users in mind, they risk failing to meet the specific requirements of people with disabilities, potentially widening health disparities (13).

In this scoping review, we aim to explore telehealth services in the UK in relation to people with disabilities. Our objectives are: (1) To examine how telehealth services are provided to people with disabilities, (2) To identify barriers, including technological and disability-specific challenges, from the perspectives of patients and healthcare providers, (3) To evaluate the experiences and views of people with disabilities and their careers regarding telehealth services, and (4) To synthesize these findings into actionable insights to guide the future design, implementation, and evaluation of telehealth services tailored to people with disabilities in the UK.

## 2 Methods

Our search methodology followed the Preferred Reporting Items for Systematic Reviews and Meta-Analyses Extension for Scoping Reviews (PRISMA-ScR). Our review protocol was not previously published. We searched the following databases: CINAHL, PubMed, Embase, Scopus and Cochrane.

The search concept ‘Telehealth’ considered all terms related to telehealth and remote consultations. Telehealth is defined as communications and information technology to provide healthcare services remotely, where the patient and provider are not in the same physical location (18). Communication modalities include real-time interactions (for example telephone or video) and asynchronous methods (for example email, text messaging, software and mobile applications) (19).

Utilising the conceptual framework of telehealth taxonomic terms by van Dyk, the term ‘telehealth’ was considered to include ‘Mobile Health (mHealth)’, ‘Telemedicine’ and ‘Telecare’ (20). Telehealth is considered to be a superset of telemedicine and related applications, such as telepharmacy, teleradiology, telepsychiatry, telecare. Under van Dyk’s conceptual framework, mHealth is considered to be a digital health technology that can contain elements from all of these areas (20). During the search process, all the subject headings under telehealth or telemedicine were included, where available. For example, the MeSH subject library included ‘Mobile Health’, ‘MHealth’, and ‘Telehealth’ under the heading ‘Telemedicine’.

The search concept ‘Disability’ is related to disability and is a synonym of impairment. Disability was defined as a physical or mental impairment that has a substantial and long-term negative effect on daily life. This was compatible with the internationally accepted definition of disability according to the International Classification of Functioning, Disability and Health (more commonly known as ICF), which includes the dimensions of impairment, activity limitations and participation restrictions (21). The type of disability was not specified or limited in order to yield as many studies as possible in the search.

Our search strategy combined the following concepts: ‘Telehealth’, ‘Disability’, ‘Impairment’, ‘United Kingdom’, and ‘NHS’. We broadened the terms for telehealth to include ‘Telemedicine’, ‘Remote’, ‘Online’, ‘Video’, ‘Telephone’ and ‘Digital Consultation’.

**Figure 1:**
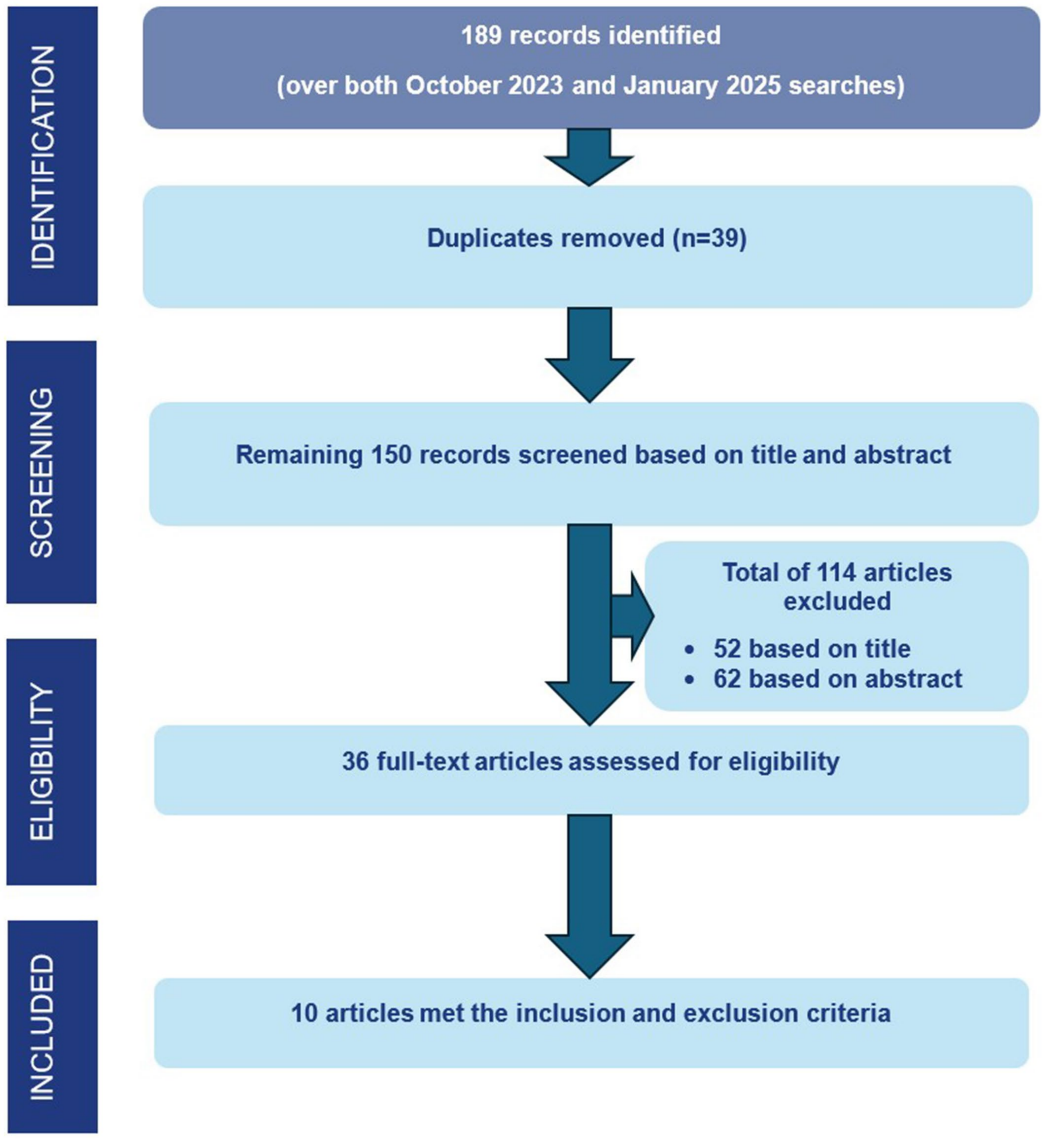
Search strategy.

Our search was initially conducted in October 2023, with another final search conducted in January 2025 to identify any new articles which had been published in the intervening period. We limited our search to studies from January 2010 onwards, supported by trend analyses of eHealth medical literature, which showed exponential growth in telehealth research after the year 2010 (22). Further relevant studies were identified from citations within papers. Our search strategy is summarized in Table 1 and Figure 1.

**Table 1:** Search strategy terminology.

Concept	Search terms
Telehealth	(exp *telemedicine/ OR exp. *telehealth/ OR E?health.mp. OR (Remote adj assess*).mp. OR (Video adj assess*).mp. OR (Video adj consult*).mp. OR (telephone adj assess*).mp. OR exp. *teleconsultation/ OR telephone consult*.mp. OR (Online adj consult*).mp. OR (Digital adj consult*).mp. OR exp. Remote Consultation/)
Disability	(exp Disabled Persons/ OR disab*.mp. OR disability/ OR exp. physical disability/ OR impair*.mp.)
United Kingdom	(exp united kingdom/ OR exp. channel islands/ OR exp. england/ OR exp. northern ireland/ OR exp. scotland/ OR exp. wales/)
National Health Service	(NHS.mp. OR “National Health Service.”mp.)

Our search strategy included the concept of ‘telehealth’ AND ‘disability’ AND (United Kingdom or National Health Service).

We included papers that presented the voice and experiences of people with disabilities in accessing and using telehealth or telemedicine services, interventional or non-interventional studies, and qualitative surveys with open-ended responses. We excluded studies conducted outside the UK, abstracts, study protocols, opinion or commentary articles, and articles involving an acute impairment or reversible condition. Two reviewers independently screened articles for inclusion (MK and YJC).

With regards to data synthesis, we used a narrative synthesis approach (23). Notes were taken during the reading of each full-text article. The quality of the studies was evaluated and discussed between the authors. Each study was categorized according to the type of study, and its key findings were summarized. Potential bias and weaknesses of the included studies were documented. While scanning the full text, patterns and themes were identified, and these findings were refined into a thematic analysis. Results were grouped and synthesized according to five different themes agreed upon by all authors: (1) Patient and carer satisfaction, (2) Benefits of telehealth (3) Health care provider perspectives (4) Disability-specific barriers, and (5) Technological barriers.

## 3 Results

A total of 189 records were identified. 39 duplicate records were manually removed. The titles of the remaining 150 records were screened for relevance to the research questions. A total of 114 articles were excluded, 52 on the basis of the title and 62 on the basis of the abstract. Following this, the remaining 36 full-text articles were assessed and reviewed to determine their relevance to the research questions of these, ten articles met both the inclusion and exclusion criteria and were included in the final analysis. Figure 1 illustrates the screening process.

The studies were characterized into the following themes: (1) Patient and carer satisfaction (*n* = 7), (2) Benefits of telehealth (*n* = 8), (3) Healthcare provider perspectives (*n* = 4), (4) Disability-specific barriers (*n* = 4), and (5) Technical barriers (*n* = 9).

The included studies employed diverse methodologies. Specifically, there were four cross-sectional surveys, one ethnographic study, one process evaluation within a randomized controlled trial (RCT), one internal pilot study within an RCT, one toolkit development and evaluation, one multi-methods evaluation with surveys and interviews, and one retrospective evaluation.

The telehealth services in the studies covered a wide range of medical specialties. Two studies focused on geriatrics, examining services for older patients with multiple comorbidities. Another three studies explored telehealth in psychiatry, specifically addressing intellectual disabilities. Two additional studies focused on neurology, one on motor neuron disease (MND) and the other on multiple sclerosis (MS). The other three single studies investigated telehealth applications in paediatrics [chronic fatigue syndrome (CFS)], audiology (tinnitus), and rehabilitation (physical disabilities).

The telehealth technologies used were also diverse. Telephone and video consultations were the primary modes of delivery in six studies. Other studies utilized specialized platforms, such as ‘Attend Anywhere’ for audiology and tinnitus care, and a custom-designed system for MND. One study employed a web-based platform to deliver cognitive behavioral therapy (CBT) for patients with CFS.

Importantly, all studies focused on delivering or evaluating telehealth services for patients with known chronic conditions. None of the included studies addressed undifferentiated diagnosis or the delivery of urgent care.

The characteristics, summary, and observations of the studies are presented in Table 2.

**Table 2:** Individual study characteristics, summary, and observations.

Publication Year	Author	Title	Journal	Study Summary	Study limitations and additional comments
2010	Bains et al. (38)	Carer satisfaction with telephone consultations	J Intellect Disabil	The majority of respondents were satisfied with telephone consultations, and were able to communicate concerns as effectively as they would have had otherwise in face-to face consultations. Despite the overall satisfaction, the majority felt that they would have preferred to see the doctor face-to-face for certain issues.	Small sample size of rural dwelling patients in a single community mental health department may introduce bias.
2013	Greenhalgh et al. (47)	What matters to older people with assisted living needs? A phenomenological analysis of the use and non-use of telehealth and telecare	Soc Sci Med	The diverse needs of patients in the study meant that successful use of assistive technologies often depended on support from family members or carers who understood the unique challenges of the patients. The study highlighted that standardized solutions frequently fail to meet the complex needs of individuals, emphasizing the importance of flexibility and adaptability of telecare and telehealth devices.	Subjectivity in both data collection and interpretation due to this being a qualitative study reflects specific context and perspectives of patients but may not be easily generalizable to other settings.
2018	Griffin et al. (36)	A questionnaire study to explore the views of people with multiple sclerosis of using smartphone technology for health care purposes	Disabil Rehabil	Explored feasibility and acceptability of smartphone use for healthcare in people with multiple sclerosis (PwMS). Smartphones were seen as beneficial with regards to improving independence and healthcare access, whilst also being time-saving. Concerns were related to reduced face-to-face contact with healthcare providers, data security, and visual impairment affecting smartphone use.	Self-selected sample recruited online might lead to selection bias.
2019	Hobson et al. (37)	Process evaluation and exploration of telehealth in motor neuron disease in a UK specialist centre	BMJ Open.	The study reported the user experience of the telehealth in MND (TiM) system. It was found to be accessible and acceptable to patients, who appreciated improved communication and coordination of care.	Study was conducted in a well-resourced tertiary specialist unit for a single disease which may limit generalisability to other settings.
2020	Anderson et al. (35)	Recruiting Adolescents With Chronic Fatigue Syndrome/Myalgic Encephalomyelitis to Internet-Delivered Therapy: Internal Pilot Within a Randomized Controlled Trial	J Med Internet Res	Explored feasibility and acceptability of recruiting adolescents with CFS into a randomised control trial comparing web-based CBT with ‘treatment as usual’ remote video consultations. Patients and their parents/caregivers found the remote nature of treatment acceptable. Patients liked tailored advice, but some families expressed preference for face-to-face treatment due to technical barriers or in the context of younger patients.	Selection bias could influence findings of the study as this study only recruited participants who were willing to undergo remote therapy.
2021	Aazh et al. (54)	Telehealth tinnitus therapy during the COVID-19 outbreak in the UK: uptake and related factors	Int J Audiol	This study explored audiologist-delivered CBT for patients with tinnitus, delivered via a video conferencing platform. Although the study did not explore patient satisfaction with the service, a survey of those who declined the service indicated reasons such as lack of a suitable device for teleconsultations, hearing loss, the belief that telemedicine would not be effective, and anxiety. Patients with worse hearing in the better ear and higher levels of tinnitus annoyance were more likely to decline.	The study did not evaluate patient satisfaction or quality of telehealth experience in comparison to face-to face therapy.
2022	Buckingham et al. (51)	Telerehabilitation for people with physical disabilities and movement impairment: development and evaluation of an online toolkit for practitioners and patients	Disabil Rehabil	Evaluation of an online resource for telerehabilitation for individuals with physical disabilities and movement impairment, with thematic analysis of the benefits and challenges experienced by users of telerehabilitation.	This study did not assess long-term outcomes of toolkit or impact of toolkit effectiveness in real-world practice.
2022	Gates et al. (40)	Telepsychiatry for people with intellectual disabilities and mental health difficulties during Covid-19 pandemic: survey of self-reported experience and acceptability to patients, carers and psychiatrists in the UK	Int J Dev Disabil	Assessed the experience and acceptability of telepsychiatry among patients, carers, psychiatrists. Showed that telepsychiatry offered benefits such as flexibility and reduced travel time. Drawbacks included loss of rapport and challenges in conducting mental state examination. While most patients reported a positive experience, 66% of patients and 69% of carers preferred face-to-face consultations.	Study was limited by low response rates for patients (24) and carers (35%).
2023	Donaghy et al. (41)	GP-led adapted comprehensive geriatric assessment for frail older people: a multi-methods evaluation of the ‘Living Well Assessment’ quality improvement project in Scotland	BJGP Open	Evaluated the use of an adapted comprehensive geriatric assessment called Living Well Assessment (LWA) conducted by general practitioners (GPs) for frail older adults. 86% of patients reported a very good experience and appreciated the holistic approach in evaluating their needs. Despite high satisfaction, most patients and carers expressed preference for face-to-face consultations, finding remote consultation difficult for building rapport.	Limited long-term outcome data on the effectiveness of LWA.
2024	Tromans et al. (39)	Acceptability of virtual psychiatric consultations for routine follow-ups post COVID-19 pandemic for people with intellectual disabilities: cross-sectional study	BJPsych Open	Evaluated the acceptability of video consultations for routine follow-up neuropsychiatric consultations of people with intellectual disabilities. There were no statistically significant differences with regards to the preference for face to face or video consultations.	The study noted potential bias from carers on participants’ response. There was limited exploration of reasons for participants’ baseline health data, and contextual factors affecting attitudes to remote consulting.

## 4 Discussion

Telehealth has long been recognized as a transformative approach with the aim of extending the reach of healthcare by overcoming geographical barriers and enhancing access to both routine and specialized services. Its potential to improve care and accessibility across various medical disciplines has been well documented (24–26). In certain circumstances, telehealth may be equivalent to or even more clinically effective than the usual standard of care, depending on the context and medical specialty (27–29).

The COVID-19 pandemic accelerated the adoption of telehealth worldwide, including the UK, where it became an essential tool for maintaining healthcare delivery during lockdown (30). The rapid adoption highlighted telehealth’s ability to bridge some health disparities, particularly for those in underserved or remote areas (24, 25). However, the evidence remains largely discipline-specific, and telehealth’s broader applications and limitations require further exploration (27, 28).

Despite these benefits, telehealth also has the potential to exacerbate existing healthcare inequities. People with disabilities experience barriers to accessing healthcare, work, public spaces and increasingly, digital technologies. Digital exclusion of vulnerable populations may limit the effectiveness of telehealth in reaching and providing healthcare for vulnerable populations, including people with disabilities (17, 24, 31–33). As a result, individuals with disabilities often face poorer health outcomes (11, 13, 34).

Given the rising adoption of telehealth, it is essential to ensure that telehealth meets the specific needs of all end-users. Our scoping review contributes to this effort, shedding light on telehealth technologies as they relate to people with disabilities.

### 4.1 Patient and carer satisfaction

Patient satisfaction was a prominent theme reported in the majority of the studies and has been generally positive. There were however nuanced challenges across different patient groups.

In the paediatric population, the web-based CBT for adolescents with CFS was well received (35). As the service was otherwise conventionally delivered from a specialized centre, many families valued the convenience of being able to avoid travel, although there were a minority (16%) who declined telehealth in favor of traditional consultations, due to technical barriers or a preference for in-person interaction.

In studies that focused on neurological conditions like MS and MND, telehealth was appreciated for improving independence and coordination of care (36, 37). However, in the case of smartphone technology for MS patients there were concerns regarding data security and a reduction in face-to-face interaction (36). For the MND study, the high number of alerts generated by the telehealth in MND (TiM) system and communication issues led to gaps between patient expectations and the responses of the health service (37).

For carers of individuals with intellectual disability, there was high satisfaction with telephone consultation, although there was still a preference for face-to face consultations (38, 39). While telepsychiatry was seen as flexible and reduced travel time, in one study a large percentage of patients and carers (66 and 69%) still preferred face-to-face consultations, due to loss of rapport and difficulties in conducting mental state examination remotely (40). In a different study conducted in 2024 on telepsychiatry, although there were no statistically significant differences between the groups who preferred face-to-face reviews to video consultations, participants who preferred face to face consultations cited a preference for being in the same room as the psychiatrist (39). A similar concern was raised in another study, where while the majority of frail older adults (86%) reported a positive experience with remote consultations, many still preferred face-to-face consultations for relationship-building and a more thorough evaluation of their needs (41).

Across the studies, the consistent theme was that telehealth offered improved access and convenience, resulting in good patient satisfaction. This was similar to results from other studies examining satisfaction with telehealth services throughout different specialties in the UK (42–44). It is still important to take a considered approach regarding what might be acceptable for patients and carers. For example, a survey of 500 patients reported that certain clinical situations are more likely to be accepted to be provided through telehealth, such as receiving and transmitting exam results and providing psychological support (45). However, the preference for face-to-face consultations remained strong, with these still considered critical for building rapport, thorough clinical examinations, and ensuring patient confidence in their care (45, 46).

### 4.2 Benefits of telehealth

Improved access to care was a central theme, which enabled timely communication for urgent concerns (38). For frail adults, comprehensive assessments of their home environment were possible without them having to leave their home, making healthcare more accessible for this vulnerable group (41). For specialised centres delivering unique services, accessibility was enhanced with the avoidance of physical and logistical challenges of having to travel to appointments (35, 37, 39).

Another key benefit was flexibility, especially in the context of persons with disabilities who might find traveling to appointments challenging (39, 40). Telecare devices also offered individuals greater independence and the ability to manage their health conditions remotely, provided that the technologies were tailored to their unique needs (47).

For the complex management of chronic diseases like MND and MS, telehealth allowed for better care coordination and monitoring (36, 37). For example, TiM system for MND improved monitoring of disease progression, with regular updates from patients enabling more responsive care.

The use of telehealth also facilitated family involvement. Family members and carers helped customise devices to fit the needs of older adults, or were integral in reporting health status (37, 47). For younger patients, their family assisted them to participate with the CBT platform (35).

Our findings mirror those of another systematic review on access to healthcare for persons with disabilities in underserved areas, where telehealth not only made it possible to access desired interventions, but also increased contact time with healthcare providers and reduced travel time and costs (44). For children and younger adults, it has also been reported that while telehealth does improve access to care, it often requires substantial support from family members or caregivers to facilitate participation (48).

### 4.3 Healthcare provider perspectives

Healthcare provider perspectives on telehealth often aligned with the concerns expressed by patients across different studies. Both groups appreciated the benefits of telehealth.

For example, general practitioners (GPs) valued the capacity for remote evaluation of frail adults, but expressed a preference for in-person visits due to difficulty in building rapport and performing thorough assessments (41). Doctors also preferred face-to-face appointments for more sensitive discussions such as ‘do not attempt cardiopulmonary resuscitation’ (DNACPR) (41). Interestingly, this specific study also raised concerns from healthcare providers regarding the time burden of this service, which required a long assessment process, and questioned its cost-effectiveness.

Communication gaps in digital interactions also frustrated both healthcare providers and patients, with the high number of alerts in the TiM system complicating effective communication (37). Mental health assessments were also challenging through a telehealth approach (40).

In line with existing literature, healthcare providers often report positive experiences with telehealth for individuals with disabilities, provided that these digital interventions deliver outcomes comparable to face-to face visits (49, 50). Reported benefits include increased patient contact time and reduced travel time and cost for patients.

A toolkit and practical guidance could serve as valuable resources for capturing and integrating both healthcare provider and patient perspectives in telehealth (51). For healthcare providers, challenges include adequate training for remote assessments, digital skills, and safety protocols – factors which are essential for the effective delivery of remote care. Co-development of such toolkits for providers with input from individuals with disabilities would also ensure that resources are both practical and adaptable to their needs, with two-way communication enhancing mutual trust.

### 4.4 Disability-specific barriers

Persons with disabilities can often present with sensory impairments which could prove particularly challenging during remote consultations (52). Persons with visual impairment often require accessible formats, such as audio instructions or screen readers, to help them navigate video conferencing platforms or mobile applications (53). For persons with sensory impairment, video consultations are generally preferred over telephone consultations as they allow for lipreading and non-verbal communication. However, even with video consultations challenges remain, especially for those who rely heavily on lipreading, since internet quality or camera angles could hinder this process (53).

We found similar barriers in our review. Patients with MS who experienced visual impairments found it difficult to navigate smartphone-based healthcare platforms, limiting their ability to fully benefit from telehealth (36). For patients with tinnitus, although the uptake of telehealth was 80%, the subgroup with more severe tinnitus and hearing loss were more likely to decline telehealth consultations due to challenges of using the video-based conferencing platform (54).

For individuals with multiple morbidities, the type of disability and its impact can be heterogenous, ranging from stroke to arthritis, unsteadiness, and incontinence. While telecare technologies allow for greater independence, the key benefit reported is the ability for devices to be customised to the needs of the individual, a term is referred to as bricolage (47, 55).

There were more barriers identified specific to patients with intellectual disability with many patients relying on carers due to limited digital literacy. Video consultations resulted in lower visibility of body language and non-verbal cues which are more critical to these group of patients. Additionally, there were potential safeguarding concerns in this vulnerable population, since video consultation might make the detection of individuals not acting in the patient’s best interests difficult.

### 4.5 Technical barriers

There were several common technical issues reported across the different studies. There were issues with poor 3G or Wi-Fi signals in some areas which caused poor video quality, or platforms to time-out while patients were still composing lengthy responses (35, 37, 40). Data security and information governance were also concerns that were faced by some patients (36, 51).

The lack of familiarity with the technology being used could affect both healthcare provider and patients (51). Difficulties in overcoming technological barriers for patients, such as setting up devices, often require support from carers or family members (40, 41). If devices or technologies are perceived as too complex or difficult to use, with poor integration into daily life and a mismatch with personal needs, patients often abandon or misuse these technologies (47).

These barriers are not unique to telehealth for persons with disabilities. A systematic review reported that commonly identified barriers to the implementation of telehealth include technically challenging staff, poor system design, bandwidth limitation, patient literacy and updated hardware (56).

Indeed, the WHO has recognized the challenge of ensuring accessibility in telehealth services and has published global standards to address this issue for persons with disabilities (57). For example, these standards stress the importance of providing adequate and specific guidance to individuals with various type of disabilities, in order to help them access telehealth services and reduce the impact of technical barriers (57).

## 5 Limitations

The studies reviewed have several limitations that may affect the generalizability and applicability of their findings. Many studies were conducted in specialist care settings (35–37, 51), often utilizing purpose-built software for teleconsultations (35, 37). While these environments provide valuable insights, they may not reflect the realities of broader healthcare systems that face disparities in resources, potentially limiting the applicability of their results to less resourced settings. One study featured a small sample size (*n* = 13), which may limit the representativeness of its results (38).

Concerns about selection bias were also noted, as many studies recruited participants who were already inclined toward remote consultations or excluded those who opted out of telehealth (35, 54). For example, one study used online recruitment, which may have resulted in findings reflecting participants who were both willing and able to access digital services (36). Understanding the reasons for this reluctance is essential to increase the acceptability of telehealth and address barriers to participation.

The concept of disability itself is multifaceted, encompassing diverse impairments and healthcare needs (11, 13). This heterogeneity necessitates a contextual application of the findings, considering the specific needs, resources, and challenges faced by different populations with disability and healthcare systems.

## 6 Conclusion

While telehealth offers numerous benefits to people with disabilities such as increased accessibility, flexibility, and independence, significant challenges remain as evidenced by this scoping review. Technological barriers such as poor internet connectivity, unfamiliarity with digital tools, and lack of appropriate devices can present difficulties. Additionally, disability-specific challenges, such as sensory impairments, further complicate the use of remote healthcare technologies.

While patient satisfaction with telehealth is generally positive, a strong preference for face-to-face consultations remains, particularly for complex medical conditions, mental health assessments, and building rapport. These findings suggest that telehealth should be viewed as complementary to traditional healthcare, through a hybrid model combining both in-person and remote care.

To fully harness the potential of telehealth for people with disabilities, future efforts should focus on improving the accessibility of technology, providing adequate training for both healthcare providers and patients, and ensuring that telehealth ecosystems are adaptable to individual needs. Tailoring telehealth solutions though co-design with patients is essential to address specific needs, reduce health disparities, and promote equitable access to healthcare.
